# Enhancer profiling uncovers Jmjd1c as an essential suppressor in neuropathic pain by targeting Socs3

**DOI:** 10.1016/j.gendis.2025.101545

**Published:** 2025-01-23

**Authors:** Le Zhang, Yan Xie, Shun Wang, Moxuan Gong, Zheping Chen, Chuanxin Wang, Peilong Li

**Affiliations:** a Department of Anesthesiology, The Second Hospital of Shandong University, Jinan, Shandong 250033, China; b Department of Clinical Laboratory, The Second Hospital of Shandong University, Jinan, Shandong 250033, China; c Shanghai Key Laboratory of Anesthesiology and Brain Functional Modulation, Translational Research Institute of Brain and Brain-Like Intelligence, Clinical Research Center for Anesthesiology and Perioperative Medicine, Department of Anesthesiology and Perioperative Medicine, Shanghai Fourth People's Hospital, School of Medicine, Tongji University, Shanghai 200434, China

**Keywords:** H3K9me1, Jmjd1c, Neuropathic pain, Socs3, Super-enhancers

## Abstract

Neuropathic pain (NP) is a chronic debilitating disease caused by nerve damage or various diseases, significantly impairs patients’ quality of life. Super-enhancers (SEs) are important cis-regulatory elements, but how they affect NP remains elusive. Therefore, we aim to explore the molecular mechanism by which SEs are involved in NP progression and identify potential drug candidate targets. We first established a NP model in rats, and subsequently performed H3K27ac ChIP-Seq and RNA-Seq on their spinal cord tissues to analyze the active enhancers. By integrated analysis of ChIP-seq data and RNA-seq data, we clarified a series of SE-associated genes involved in NP progression. qPCR and double immunofluorescence staining results suggested that *Jmjd1c* mRNA and protein levels were significantly down-regulated in the NP model. In addition, a dual-luciferase reporter assay showed that *KLF15* could activate *Jmjd1c* transcription by binding to the SE of *Jmjd1c*. Functionally, enhanced *Jmjd1c* can inhibit the levels of inflammatory cytokines such as IL-6, TNF-α, IL-1β, and inhibited the progression of NP, whereas silencing *Jmjd1c* had the opposite effect. Mechanistic exploration identified *Jmjd1c* exerted its anti-NP effect via positively regulating *Socs3* expression by increasing the activity of H3K9 demethylation, and the *Jmjd1c/Socs3/JAK/STAT3* regulatory pathway was finally validated as downstream effectors. In conclusion, our study suggests that SE-associated *Jmjd1c* was suppressed during NP progression due to the decreased recruitment of *KLF15*. The reduction of *Jmjd1c* downregulated *Socs3* through the demethylation of H3K9 at *Socs3* promoter region, leading to NP progression.

## Introduction

Neuropathic pain (NP) is the most challenging neurological disease worldwide, with population-based estimates of its prevalence varying from 7% to 10%,^1^ and most patients suffer from an impaired quality of life. The main signs and symptoms of NP include spontaneous pain, allodynia, and hyperalgesia.^2^ A general consensus is that specific pathological causes of NP include spinal cord or peripheral nerve injury, postherpetic neuralgia, trigeminal neuralgia, and tumor invasion.^3^ The increased excitability of spinal dorsal horn neurons following nerve damage is a crucial factor in the onset and maintenance of NP. Additionally, the overproduction of inflammatory cytokines contributes to this excessive activation of spinal dorsal horn neurons.^4^ Despite significant efforts by scientists, the specific molecular mechanisms regulating glial activation and cytokine production in NP patients are not fully understood. Therefore, further understanding of the specific molecular mechanisms is of significant interest for exploring therapeutic targets and developing treatments to improve the quality of life for NP patients.

Mounting evidence supports the potential role of epigenetic regulators in different types of diseases.^5^ Typical enhancers are short cis-regulatory DNA region elements in the genome, ranging from 50 to 1500 bp. These elements could bind to transcriptional regulatory proteins and activate target gene transcription via long-range cis-chromatin interactions.^6^^,^^7^ Besides typical enhancers, the genome also harbors multiple consecutively arranged enhancers with distances between 8 and 20 kb, referred to as super-enhancers (SEs).^8^ However, SEs usually recruit more transcription factors, histone-modifying enzymes, transcriptional co-activators, and RNA polymerases than typical enhancers to drive the transcription of target genes, thereby exerting a powerful regulatory effect on cell functions.^9^ Although studies have reported that SEs can regulate NP progression via driving the transcription of genes such as N-terminal Xaa-Pro-Lys N-methyltransferase 1 (*Ntmt1*) and paired related homeobox 2 (*Prrx2*).^10^ Identification of dysregulated SEs involved in the pain signaling pathway remains incomplete, and downstream validation of the functional SEs is scarce.

Suppressor of cytokine signaling 3 (*Socs3*) is an important inflammatory mediator that negatively regulates the signal transduction pathways of cytokines in a variety of cell types, including immune and central nervous system cells.^11^^,^^12^ The Janus kinase (*JAK*)-signal transducer and activator of transcription (*STAT*) pathway, evolutionarily conserved, is activated by cytokine stimulation and appears to be a crucial factor in a variety of key physiological processes.^13^ Evidence has shown that exosomal miR-222-3p regulates the *JAK2-STAT3* pathway via promoting *Socs3* encoding an inhibitory factor in gemcitabine-resistant cells.^14^ Thus, we supposed that *Socs3* might regulate pain response in NP via regulating *STAT3* activation. However, it remains unclear whether and how the specific molecular mechanism of nerve cell response and adaptive behavior to pain cytokine infiltration depends on the intrinsic characteristic referring to the SEs of activity.

Here, we aim to reveal the SE landscape of NP, which could provide a theoretical basis for the in-depth understanding of the molecular pathogenesis underlying pain responses in neurons, and offer data support for exploring potential new therapeutic targets. Therefore, we performed H3K27ac chromatin immunoprecipitation sequencing (ChIP-seq) and the corresponding RNA sequencing (RNA-seq) in spared nerve injury (SNI)-induced NP rats and sham rats to characterize NP-related transcriptomic and epigenomic changes. We subsequently found that a reduction in the transcription factor *KLF15* (KLF transcription factor 15) recruited by the Jumonji domain-containing 1C (*Jmjd1c*)-SE led to lower levels of *Jmjd1c* expression, which in turn facilitated pain responses in neuronal cells both in *vitro* and in *vivo*. Mechanistically, *Jmjd1c* collectively promoted the transcriptional activity of *Socs3* by promoting H3K9 demethylation on the *Socs3* promoter, thereby affecting the *JAK-STAT3* pathway and leading to an anti-NP effect. Overall, these results help to deepen our knowledge of how SEs play a functional role during NP progression and provide new therapeutic targets for NP.

## Materials and methods

### Animals

In this study, all healthy male Sprague–Dawley (SD) rats (aged 8–10 weeks, weighing 180–200 g) were obtained from the Model Animal Research Center of Nanjing University. These rats were housed and maintained in standard laboratory conditions, which featured a regulated 12-h/12-h light/dark cycle at 24 °C ± 1 °C, with lights on from 8:00 a.m. to 8:00 p.m., and a humidity of 40%–50%.

### Pain model construction and treatment

As previously described,^15^^,^^16^ an SNI rat model was used to induce and simulate the process of NP, with rats undergoing sham surgery serving as controls. In accordance with our experimental protocol, the SD rats were allocated to ten distinct groups and then underwent various treatments according to the experimental objectives: i) Sham, ii) Sham+sh-*Jmjd1c*, iii) Sham+sh-NC, iv) Sham+sh-*Jmjd1c*+Lv-Socs3, v) Sham+sh-*Jmjd1c*+Lv-NC, vi) SNI, vii) SNI+Lv-*Jmjd1c*, viii) SNI+Lv-NC, ix) SNI+Lv-*Jmjd1c*+sh-Socs3, x) SNI+Lv-*Jmjd1c*+sh-NC. The spontaneous pain behaviors were measured at corresponding time points, and the expression level of inflammatory factors in spinal cord tissue was measured after euthanasia. All animal experiments in our study were rigorously examined and approved by the Animal Experiment Committee of The Second Hospital of Shandong University (KYLL-2021(KJ)A-0294) and strictly adhered to the standards set by the International Association for the Study of Pain.

### RNA-seq

We used the commercially available TRIzol reagent (Invitrogen, USA) to extract total RNA from the spinal cord tissues of SD rats. Subsequently, the RNA-seq library was generated and sequenced using an Illumina HiSeq 3000 system by GENE DENOVO (Guangzhou, China). The obtained sequences were mapped against the Rat6 reference genome, and only those unique sequences were kept. Fold change of the gene's reads per kilobase million (FPKM) was used to identify differentially expressed genes using DESeq2. The original sequencing data were deposited in the GEO database (GSE256472).

### H3K27ac ChIP-seq

The SD rats were rendered deeply unconscious using appropriate concentrations of isoflurane, after which their spinal cord tissues were obtained and fixed with 1% formaldehyde. Nuclear chromatin was extracted and segmented into 100–400 bp fragments by ultrasound. A 5% aliquot of the fragmented chromatin was set aside as a reference sample, while the rest was subjected to incubation with a ChIP-grade anti-H3K27ac antibody. Chromatin immunocomplexes were then decross-linked with RNase A (NEB, T3018L) and proteinase K (NEB, P8107S). After washing the immunoprecipitated complexes, DNA extraction and purification were carried out utilizing a PCR purification kit (Qiagen, 28106). Subsequently, ChIP-seq libraries were prepared from the purified DNA, and next-generation sequencing was performed using Novaseq PE150 by Shanghai Jiayin Biotechnology Ltd. The original sequencing data were deposited in the GEO database (GSE256469).

### Super-enhancer definition

The clean H3K27ac ChIP-seq data were aligned to the Rat6 genome assembly available in Ensembl using Burrows-Wheeler Aligner with default parameters. Subsequently, MACS2 (version 2.2.7) was utilized to identify the H3K27ac peak calling regions, setting an initial threshold q-value of 0.05. H3K27ac-rich regions within ±2 kb of the transcription start site were first filtered out, and the remaining regions were considered enhancers. Then, enhancers located within a 12.5 kb range were combined, and they were arranged in order of the intensity of the ChIP-seq signal. Then, a plot was graphed and a tangent line of the curve was determined with the slope value of 1, SE was identified as the enhancer above the point of tangency.

### Integration analysis of ChIP-seq and RNA-Seq

To uncover the functional epigenetic modification and potential downstream target genes that they may regulate as accurately as possible, we conducted a comprehensive analysis by combing data from H3K27ac ChIP-seq and RNA-seq. In cases where a gene corresponds to multiple ATAC-seq peaks, we chose the peak with the highest signal intensity near that gene. Similarly, when a ChIP-seq peak is associated with multiple genes, we choose the gene nearest to the peak for association. Genes that showed significant up-regulation or down-regulation in the RNA-seq analysis were compared and matched with those exhibiting increased or decreased ChIP-seq signal intensities in the H3K27ac-enriched regions. The enrolled analyzed SEs were chosen based on fold change ≥ 1 between SNI rats and sham rats, while the analyzed RNAs were chosen based on fold change ≥ 1.2 between the two groups.

### NP behavior assessment

To assess the degree of mechanical allodynia, we measured the paw-withdrawal threshold (PWT) in response to stimulation with von Frey filaments. SD rats were placed in a transparent plastic box implanted with metal receptors (22 × 12 × 22 cm) and were habituated to the environment for at least 30 min until they stopped exploring and grooming. Pain sensitivity was ascertained by the minimum filament force required to provoke a withdrawal reaction in at least three out of five attempts. The measurement was taken once the paw withdrawal was clearly recognized. Additionally, we tallied the number of spontaneous flinches (NSF) over a 2-min interval to evaluate the degree of spontaneous pain. Any unprovoked lifting of the right hind paw that was not part of walking or grooming was accounted for as a spontaneous flinch.

### Enzyme-linked immunosorbent assay (ELISA)

Tissues from the spinal cords of adult SD rats were isolated, followed by centrifugation at 10,000 rpm for 10 min to separate the supernatant. Subsequently, the collected supernatants were preserved at −80 °C until use. Commercial ELISA kits for quantification of the cytokines interleukin (IL)-6, tumor necrosis factor-alpha (TNF-α), and IL-1β were purchased from Multi Sciences (Lianke, China). The analyses were conducted following the manufacturer's protocol for each ELISA kit, the absorbance was read at 450 nm with a microplate reader (SpectraMax M3) to assess the concentration of cytokines.

### Cell culture

The rat neuronal-spinal cord (RN-sc) cell line, obtained from ScienCell (Catalog #R1590), was grown in a neurobasal medium supplemented with 10% fetal bovine serum. The cells were cultured in an incubator maintained at 37 °C and a humidified atmosphere containing 5% CO_2_. For the transfection *in vitro*, Lipofectamine 2000, a mature commercial cationic liposome transfection reagent, was used to modulate the expression of specific genes in RN-sc cells. The sequences of the silencing vectors used are detailed in Table S1.

### Intrathecal catheter implantation and lentivirus injection

Lentiviral vectors incorporating specific oligonucleotides (GenePharma, China) were constructed to stably manipulate the expression of *Jmjd1c*. The implantation of the intrathecal cannula was carried out following a previously described protocol.^17^ Briefly, under isoflurane inhalation anesthesia, the lumbar region of rats was disinfected with 75% ethanol after shaving the hair. A polyethylene tube (PE-10) was carefully placed into the epidural space between the fifth and sixth lumbar vertebrae (L5–L6). Once the rats had fully recovered from anesthesia, the correct positioning of the catheter was verified by observing whether the injection of 0.2 mL of a 2% lidocaine solution resulted in signs of dragging or paralysis in the hind limbs. Only rats that showed no evidence of neurologic deficit or paralysis were studied. A comprehensive timeline of the treatment process is presented in Figure S1.

### Luciferase reporter assay

The enhancer segments E1, E2, and E3 from the *Jmjd1c*-SE region were inserted into the pGL3-Basic vector (BioVector NTCC Inc.). Concurrently, an empty pGL3-basic vector was utilized to establish a baseline luciferase activity, serving as a negative control. RN-sc cells were seeded at a concentration of approximately 1 × 10^5^ cells per well in a 24-well plate and then transfected with 100 ng of the luciferase vectors (either the empty vector or those containing the enhancer segments E1, E2, and E3) along with 10 nM of RNA interference (sh-control or sh-KLF15) using Lipofectamine 2000 reagent. After a 48-h incubation period following transfection, treated cells were lysed, luciferase substrate was added, and fluorescence values were measured according to the protocols of the Dual-Luciferase Reporter Analysis System (Promega, USA). The luciferase activity was quantified as the ratio of Firefly to Renilla (Firefly/Renilla) and was normalized to the activity of the cells transfected with the empty pGL3-Basic vector.

### Quantitative PCR (qPCR)

Briefly, we extracted total RNA from L4–L6 spinal cord tissues and RN-sc cells and then converted it into cDNA using a commercial cDNA synthesis kit. Subsequently, we performed qPCR with SYBR Green PCR Master Mix (Takara, China) to detect specific gene expression, utilizing a QuantStudio 3 system (Thermo Fisher, USA) for quantitative analysis. The 2^−ΔΔCt^ approach was applied to calculate the relative mRNA expression levels as previously described,^18^ with GAPDH serving as a reference gene. The primers utilized in this study are detailed in Table S2.

### ChIP-qPCR

A ChIP-qPCR study was carried out on samples from RN-sc cells and SNI rats to evaluate *KLF15* binding in the E2 and E3 elements of *Jmjd1c*. Briefly, 1 × 10^7^ nerve cells were cross-linked with formaldehyde at a final concentration of 1 %, and then the cells were subjected to sonication using a Diagenode Bioruptor to generate DNA fragments in the 200–500 bp size range, followed by immunoprecipitation using antibodies specific to *KLF15* (Abcam, ab2647) and a control normal immunoglobulin G (IgG) (CST, 2729S). RNase A and protease K were employed to break down the RNA and protein components of the protein-DNA complexes, respectively. Immunoprecipitated DNA fragments were measured by qPCR. Similarly, the binding enrichment of *Jmjd1c* and H3K9me1 at the *Socs3* promoter was also conducted as above described. Detailed information on antibodies used is provided in Table S3.

### Immunofluorescence staining analysis

In our study, we used immunofluorescence to determine the levels of *Jmjd1c* expression and its co-localization with other proteins in spinal cord samples from SD rats. Briefly, we treated the frozen coronal sections with primary antibodies and incubated them in the dark at 4 °C. Subsequently, these sections were further incubated with the corresponding fluorescently labeled secondary antibodies at room temperature for 2 h. Detailed information on antibodies used is provided in Table S3. Finally, the image of stained tissues was obtained using a Laser confocal scanning microscope (Olympus, Japan) in the dark room.

### Western blotting

After total protein was extracted from cells or tissues and its concentration was determined, appropriate amounts of protein samples were loaded onto an SDS polyacrylamide gel for electrophoretic separation. These proteins were then transferred onto a PVDF membrane and pre-treated with a 5% solution of bovine serum albumin to prevent non-specific binding, after which they were incubated with their respective primary antibodies at 4 °C within a dark chamber overnight. After being rinsed with tris buffered saline with Tween 20, they are exposed to horseradish peroxidase-linked secondary antibodies at room temperature for 1 h. Detailed information on the antibodies used in western blotting is provided in Table S4. Finally, protein bands were detected utilizing enhanced chemiluminescence and their images were visualized using an imaging system (Tanon, China).

### Statistical analysis

In most of our analyses, GraphPad Prism 9 (GraphPad Software, USA) for Windows was used to carry out statistical evaluations and to create graphical representations. Unless specified otherwise, the data were expressed as mean ± standard error of the mean. When comparing parametric variables between two groups, the student's *t*-test was utilized when variances were equal, and two-tailed distributions were used. In cases involving non-parametric variables from two distinct samples, the Mann–Whitney *U* test was used. A *P*-value of less than 0.05 was considered statistically significant.

## Results

### Enhancer landscapes in spinal cord tissues of rats with NP

**Figure 1:**
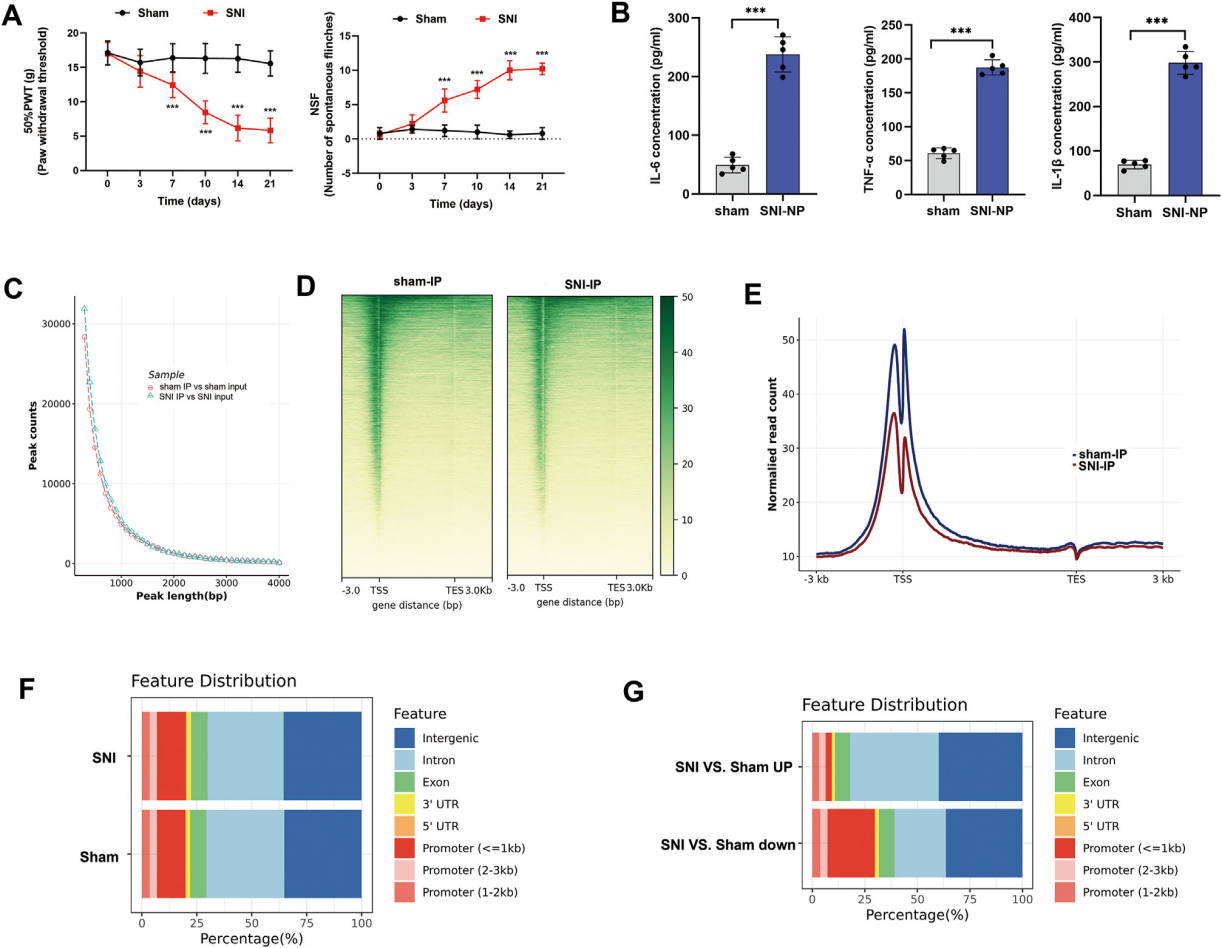
Enhancer landscapes in spinal cord tissues of rats with NP. **(A)** Experimental validation of NP rat model by detecting PWT and NSF. **(B)** The expression levels of IL-6, TNF-α, and IL-1β were detected by ELISA in respective groups. **(C)** The counts of peak length by H3K27ac-based chromatin immunoprecipitation sequencing were calculated in both groups. **(D)** The landscape of the H3K27ac enrichment profile surrounding the transcription start site in SNI-NP and sham rat tissues. **(E)** The abundance of reads surrounding TSS and TES was calculated in SNI and sham rats. **(F)** Calculation and distribution of H3K27ac enriched reads in genome area. **(G)** Genomic distribution of differentially enriched peaks between SNI-NP and sham rats. The statistical results were presented as mean ± standard deviation. Student's *t*-test was used to determine the significance of the difference between the two groups. The asterisks indicate significance values (^∗∗∗^*P* < 0.001). NP, neuropathic pain; PWT, paw-withdrawal threshold; NSF, the number of spontaneous flinches; SNI, spared nerve injury; ELISA, enzyme-linked immunosorbent assay; TSS, transcription start site; TES, transcription end sites.

Rats were randomly divided into two groups. One group underwent the SNI procedure to mimic the NP status, while the other group received a sham procedure and served as controls. Before the experiment was started, both groups showed similar PWT and NSF, indicating no significant baseline differences in these pain-related parameters. However, decreased PWT and increased NSF were verified after the SNI-NP model was established, but not in sham rats (Fig. 1A). Concurrently, ELISA results revealed increased levels of cytokines, including IL-6, TNF-α, and IL-1β, in the spinal cord tissue of SNI-NP rats (Fig. 1B). We performed ChIP-seq analysis using H3K27ac antibody, which reflect the enhancer activity of critical genes involved in NP. A total of 153,276 and 138,940 peaks were identified in SNI-NP tissues and sham tissues, respectively. Peak length calculation suggested a consistent distribution between SNI-NP and sham rats (Fig. 1C). H3K27ac marks exhibited a profile surrounding the transcription start site (Fig. 1D). Interestingly, the abundance was lower in SNI-NP group compared with sham group (Fig. 1E). The analysis of peak annotations indicates that the H3K27ac modification predominantly occurs in promoters (19.79%), introns (35.57%), and distal intergenic regions (35.14%) within the spinal cord of SNI-NP rats, consistent with that in sham rats (Fig. 1F). By comparing the peaks between the two groups, we identified 9946 up-regulated and 7376 down-regulated peaks in the SNI-NP group in contrast to the sham group, with criteria of |log2 fold change| > 1 and *P* < 0.05. Genome distribution revealed that the differential peaks were mainly located in intergenic and intron areas (Fig. 1G). Interestingly, a proportion of down-regulated peaks in the NP group was located in the promoter area, suggesting a group of genes may be silenced during NP development (Fig. 1G).

### Characterization of SE landscape in the spinal cord of SNI-NP rats

**Figure 2:**
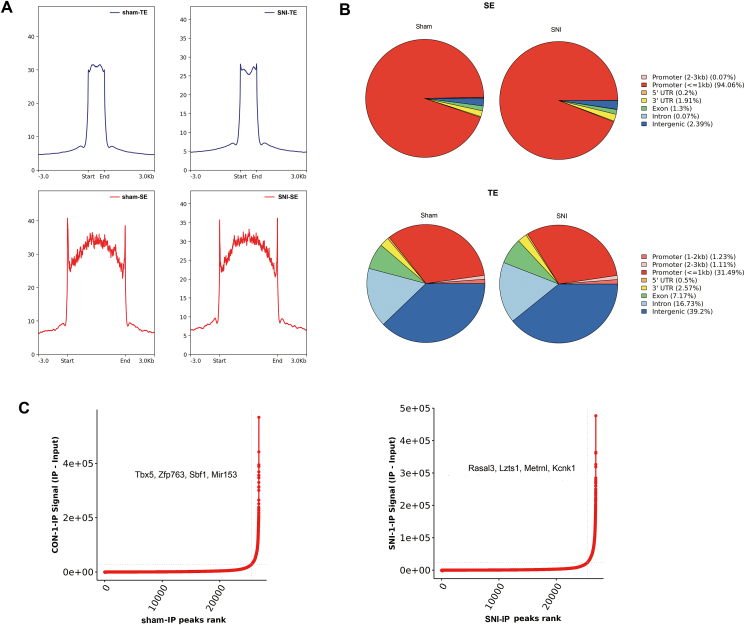
Super enhance identification in the spinal cord of NP rats. **(A)** The signal distribution of peaks in SEs and TEs. A stronger and wider signal content was identified. **(B)** Genomic distribution of SEs and TEs was significantly different, with SEs located at the promoter area while TEs at the intergenic area. **(C)** ROSE analysis revealed a series of super-enhancers in both NP and sham groups. NP, neuropathic pain; SEs, super-enhancers; TEs, transposable elements.

We conducted a ROSE (rank ordering of super-enhancers) analysis to identify particular SE regions in SNI-NP rats. The signal enriched in SE was significantly stronger and wider than typical enhancers (Fig. 2A). Totally, we annotated 1265 and 1464 SEs significantly enriched in SNI-NP and sham rats, respectively. Functional area analysis revealed that SEs in both groups were mostly localized in the promoter area, while typical enhancers were located in intergenic areas, suggesting a powerful role of SEs in gene transcription (Fig. 2B). A group of potential NP-related genes was identified located nearby SEs, including *Rasal3*, *Lzts1*, *Metrnl*, and *Kcnk1* (Fig. 2C).

### Identification of SE-associated *Jmjd1c* in NP progression

**Figure 3:**
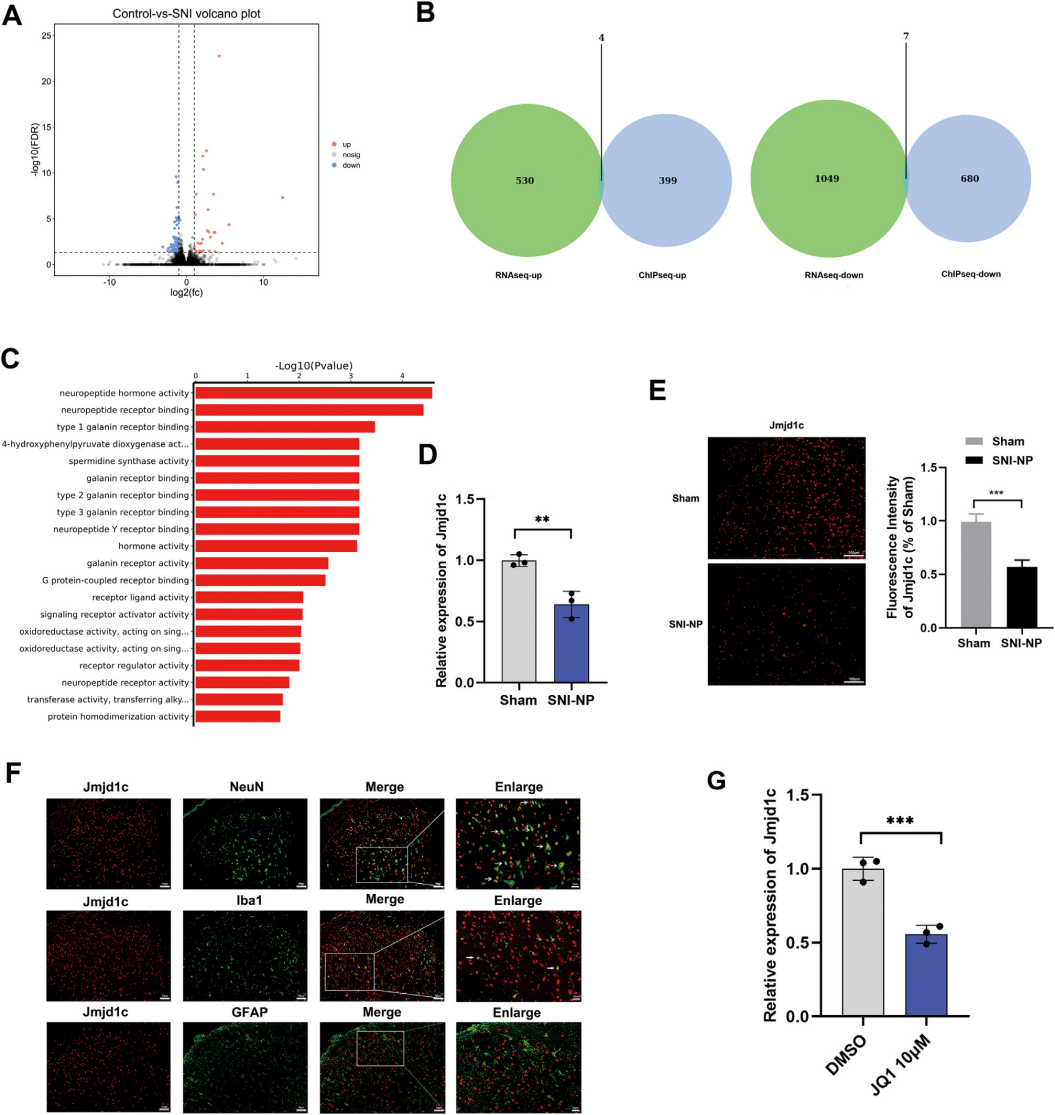
*Jmjd1c* is regulated by SEs and located at neuron cells. **(A)** The volcano plot showed the differential expressed RNAs between tissue samples from NP rats and sham rats. **(B)** Integrated analysis of chromatin immunoprecipitation sequencing data and RNA sequencing data yielded a total of 11 differentially expressed super-enhancer-related genes, including four up-regulated genes and seven down-regulated genes. **(C)** GO analysis of the SE-associated RNAs revealed an enrichment of neuropeptide-related regulation. **(D)** The expression of *Jmjd1c* mRNA was detected by quantitative PCR. **(E)** The *in-situ* expression of *Jmjd1c* protein was detected via immunofluorescence. **(F)** Dual immunofluorescence staining showed the expression of *Jmjd1c* in neuron cells, microglial cells, and astrocyte cells, respectively. **(G)***Jmjd1c* expression levels in human RN-sc cells treated by JQ1 were measured by quantitative PCR. The statistical results were presented as mean ± standard deviation. Student's *t*-test was used to determine the significance of the differences between the two groups. The asterisks indicate significance values (∗∗*P* < 0.01, ^∗∗∗^*P* < 0.001). NP, neuropathic pain; GO, gene ontology; SEs, super-enhancers.

To reveal the SE-associated genes involved in NP, we performed RNA-seq analysis using spinal cord tissues from NP and sham rats. Based on our analysis, a total of 106 differentially expressed mRNAs were determined by the criteria of a fold change greater than 2 and a *P*-value less than 0.05. Among these, 31 were up-regulated and 75 were down-regulated in NP rats compared with sham rats, such as nervous system disease-related genes *Zeb1*, *Atf3*, *Vip*, and *Aurkb* (Fig. 3A). By integrative analysis with ChIP-seq data, we identified 11 potential SE-associated genes, including 4 up-regulated (*Per3*, *Antxr1*, *Pdlim1*, and *Ccdc40*) and 7 down-regulated ones (*AABR07061825.1*, *Chd9*, *Ank2*, *Jmjd1c*, *Dnajc28*, *Zeb1*, and *Zfp462*) (Fig. 3B). GO analysis revealed that neuropeptide-related regulation may play essential roles in NP progression (Fig. 3C). Candidate SE-associated genes were screened based on the principles of primer design, gene expression levels, and the consistency of differential expression. Among the 11 genes, specific primers could not be designed for genes *AABR07061825.1*. Additionally, the expression level of *Per3* was too low to meet the requirements for PCR detection. For the remaining candidate genes, qPCR analysis revealed that *Jmjd1c*, *Ccdc40*, and *Dnajc28* exhibited a change in expression in the spinal cord of SNI-NP rats, consistent with the sequencing results. Among these three candidate genes, *Jmjd1c* was reported to be involved in neurological disorders. Thus, we focused on the role of *Jmjd1c* in SE-regulated NP (Fig. 3D; Fig. S2). Immunofluorescence analysis further validated that *Jmjd1c* protein was down-regulated in NP rats (Fig. 3E). Dual immunofluorescence staining results showed that *Jmjd1c* was primarily expressed in the NeuN (neuron cell)-positive cells, partially expressed in the lba1 (microglial cell)-positive cells and negatively expressed in the GFAP (astrocyte cell)-positive cells (Fig. 3F). Next, we utilized JQ1, which inhibits *BRD4*, to interfere with the activity of SEs. As a result, a marked decrease in *Jmjd1c* expression was observed in RN-sc cells exposed to JQ1 (Fig. 3G). The above results suggest that *Jmjd1c* may be a key gene associated with SE in the progression of NP.

### *KLF15* activates *Jmjd1c* transcription by binding to its SE

**Figure 4:**
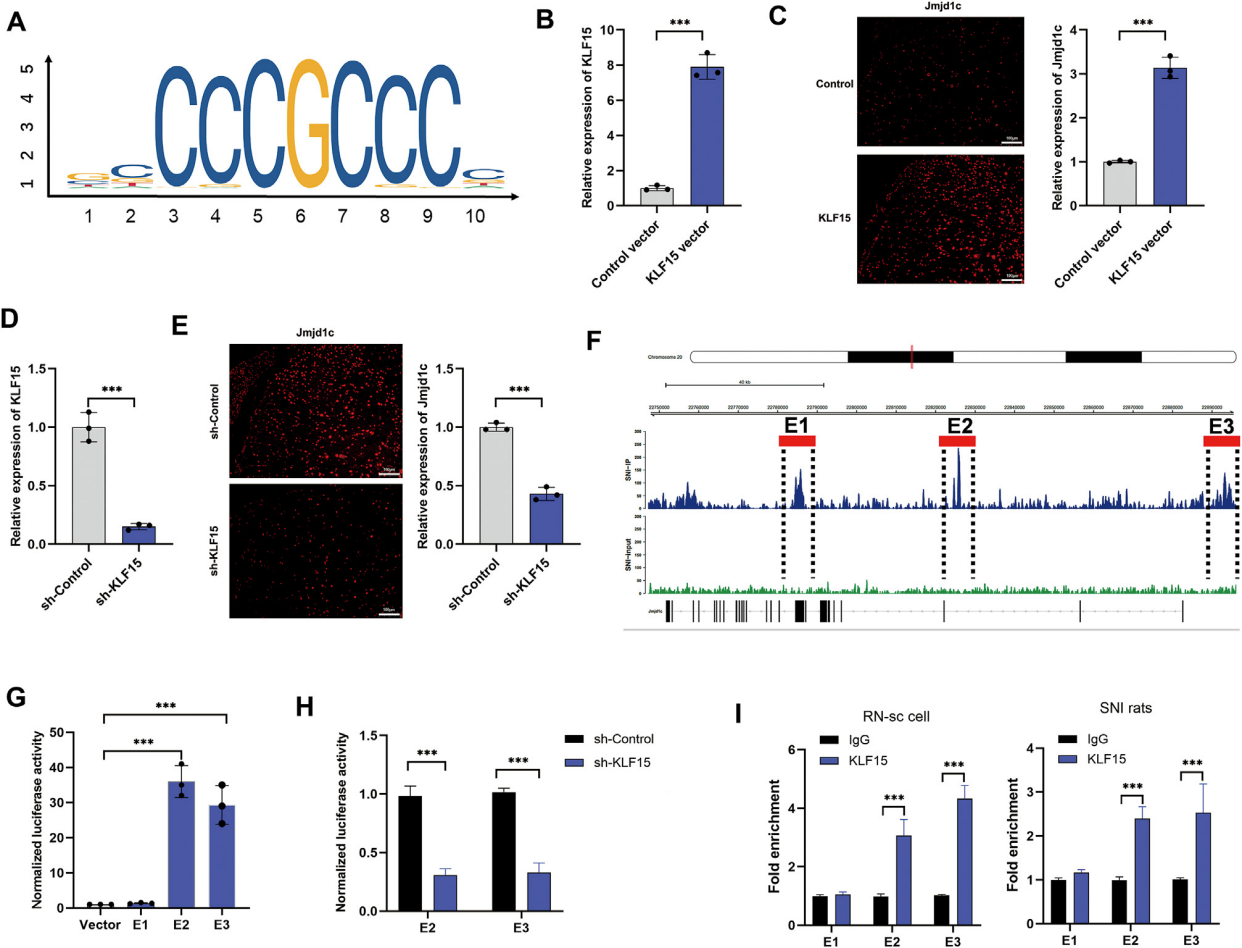
*KLF15* enriches at *Jmjd1c*-SE and promotes the expression of *Jmjd1c*. **(A)** The binding site motif of transcription factor *KLF15*. **(B)** Quantitative PCR was performed to detect the overexpression efficiency of *KLF15* in RN-sc cells. **(C)** Immunofluorescence was used to detect the protein level of *Jmjd1c* upon overexpression of *KLF15*. **(D)** Quantitative PCR was used to detect the knockdown efficiency of *KLF15* in RN-sc cells. **(E)** Immunofluorescence detection showed the expression of *Jmjd1c* after knocking down *KLF15* expression. **(F)** Genome browser tracks showed three potential binding regions of *KLF15* and *Jmjd1c*. **(G)** RN-sc cells were co-transfected with three potential KLF15-specific elements (E1, E2, and E3) and *Jmjd1c*, and luciferase activity was determined using a dual-luciferase assay system. **(H)** Dual-luciferase assay showed the luciferase activity changes of KLF15-specific elements (E2 and E3) in RN-sc cells after knocking down *KLF15*. **(I)** Chromatin immunoprecipitation-quantitative PCR was performed on RN-sc and SNI rats to evaluate the binding ability of *KLF15* to E1, E2, and E3 elements of *Jmjd1c*-SE. The statistical results were presented as mean ± standard deviation. Student's *t*-test was utilized to evaluate the differences between the two groups. The asterisks indicate significance values (^∗∗∗^*P* < 0.001).

Given that SEs frequently recruit core transcription factors to activate the target gene transcription, we sought to find the potential transcription factors. Among the predicted transcription factors, we focused on *KLF15*, which was reported to participate in pain-related diseases^19^ (Fig. 4A). By generating *KLF15* specific overexpressing and silencing vectors, we proved that *KLF15* positively regulated *Jmjd1c* expression at both transcript and protein levels (Fig. 4B–E). To determine how *KLF15* contributed to the activity of *Jmjd1c*-SE, we inserted three *KLF15*-specific DNA sequences (E1, E2, and E3) into the pGL3-Basic plasmid (Fig. 4F). A luciferase reporter assay was performed to quantify their enhancer activities, and the results revealed that E2 and E3, but not E1, exhibited significantly elevated enhancer activities compared with the control vector (Fig. 4G). Importantly, knocking down *KLF15* expression in RN-sc cells led to a reduction in the enhancer activities of E2 and E3 (Fig. 4H). In addition, the ChIP-qPCR assay further confirmed that *KLF15* was enriched in E2 and E3 elements, but not in E1 element in both RN-sc cells and SNI rats (Fig. 4I).

### *Jmjd1c* suppress NP progression in SNI rat model

**Figure 5:**
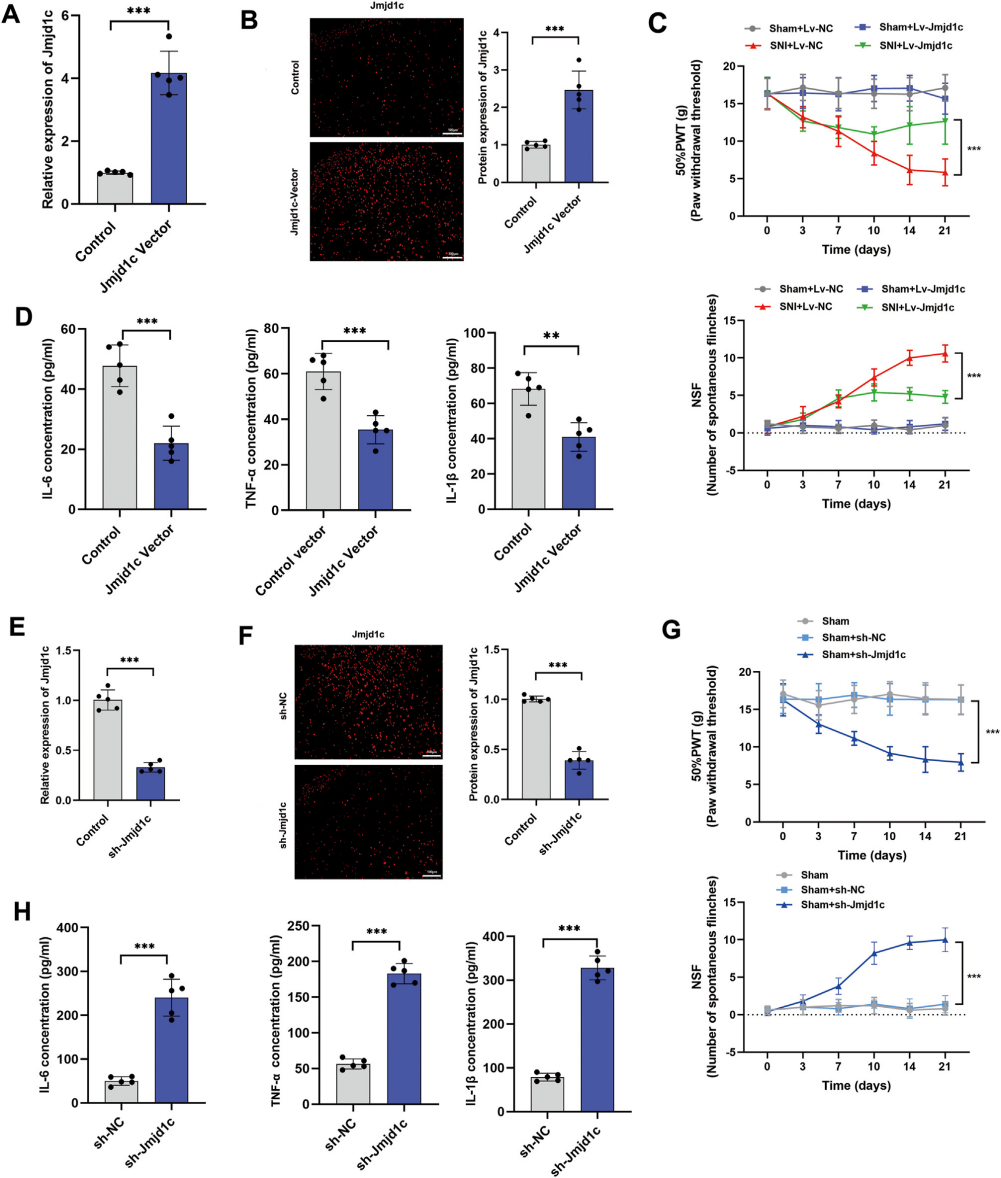
*Jmjd1c* plays a suppressive role in NP progression. **(A)** Quantitative PCR showed that the relative expression of *Jmjd1c* mRNA was increased in *Jmjd1c*-overexpressing rats. **(B)** Immunofluorescence detection showed that the protein expression of *Jmjd1c* was increased in *Jmjd1c*-overexpressing rats. **(C)** PWT (upper) and NSF (lower) analyses indicated that *Jmjd1c* overexpression significantly alleviated the symptoms of NP in SNI rats, but showed no effect on sham rats. **(D)** ELISA was performed to detect the expression levels of IL-6, TNF-α, and IL-1β in SNI rats overexpressing *Jmjd1c*, and a significantly decreased expression of these markers was identified. **(E)** Quantitative PCR detection showed the relative expression changes of the *Jmjd1c* in Jmjd1c-overexpressing rats. **(F)** Immunofluorescence detection showed that the protein expression of *Jmjd1c* was decreased after knocking down *Jmjd1c* expression. **(G)** PWT (upper) and NSF (lower) analyses showed that silencing of *Jmjd1c* significantly induced the NP in sham rats. **(H)** The expression of inflammatory markers (IL-6, TNF-α, and IL-1β) was detected via ELISA, and the expression of three markers was significantly increased in *Jmjd1c*-silencing sham rats. The statistical results were presented as mean ± standard deviation. Student's *t*-test was used to evaluate the difference between the two groups. The asterisks indicate significance values (^∗∗∗^*P* < 0.001). NP, neuropathic pain; PWT, paw-withdrawal threshold; NSF, the number of spontaneous flinches; SNI, spared nerve injury; ELISA, enzyme-linked immunosorbent assay.

To verify the functional significance of *Jmjd1c*, we designed oligonucleotides mimicking *Jmjd1c* and overexpressed *Jmjd1c* via intrathecal administration of SNI rats at time point as indicated by the flow chart (Fig. S1). As shown, after intrathecal injection of the *Jmjd1c* mimic, the expression levels of *Jmjd1c* were markedly elevated in rat spinal cord tissues at both transcript and protein levels (Fig. 5A, B; Fig. S3A). Moreover, enhanced *Jmjd1c* elevated PWT and suppressed NSF in SNI rats (Fig. 5C). Consistently, the expression levels of the three cytokines were also suppressed after injection of *Jmjd1c* vector (Fig. 5D). On the other hand, we silenced *Jmjd1c* in sham rats by intrathecal injection of respective silencing vector (Fig. 5E, F; Fig. S3B). The results showed that suppression of *Jmjd1c* promoted NP behavior, including suppressed PWT and increased NSF (Fig. 5G). Meanwhile, related inflammatory factors, such as IL-6, TNF-α, and IL-1β were also significantly up-regulated according to ELISA results (Fig. 5H).

### *Jmjd1c* suppressed NP via positive regulation of *Socs3* expression

**Figure 6:**
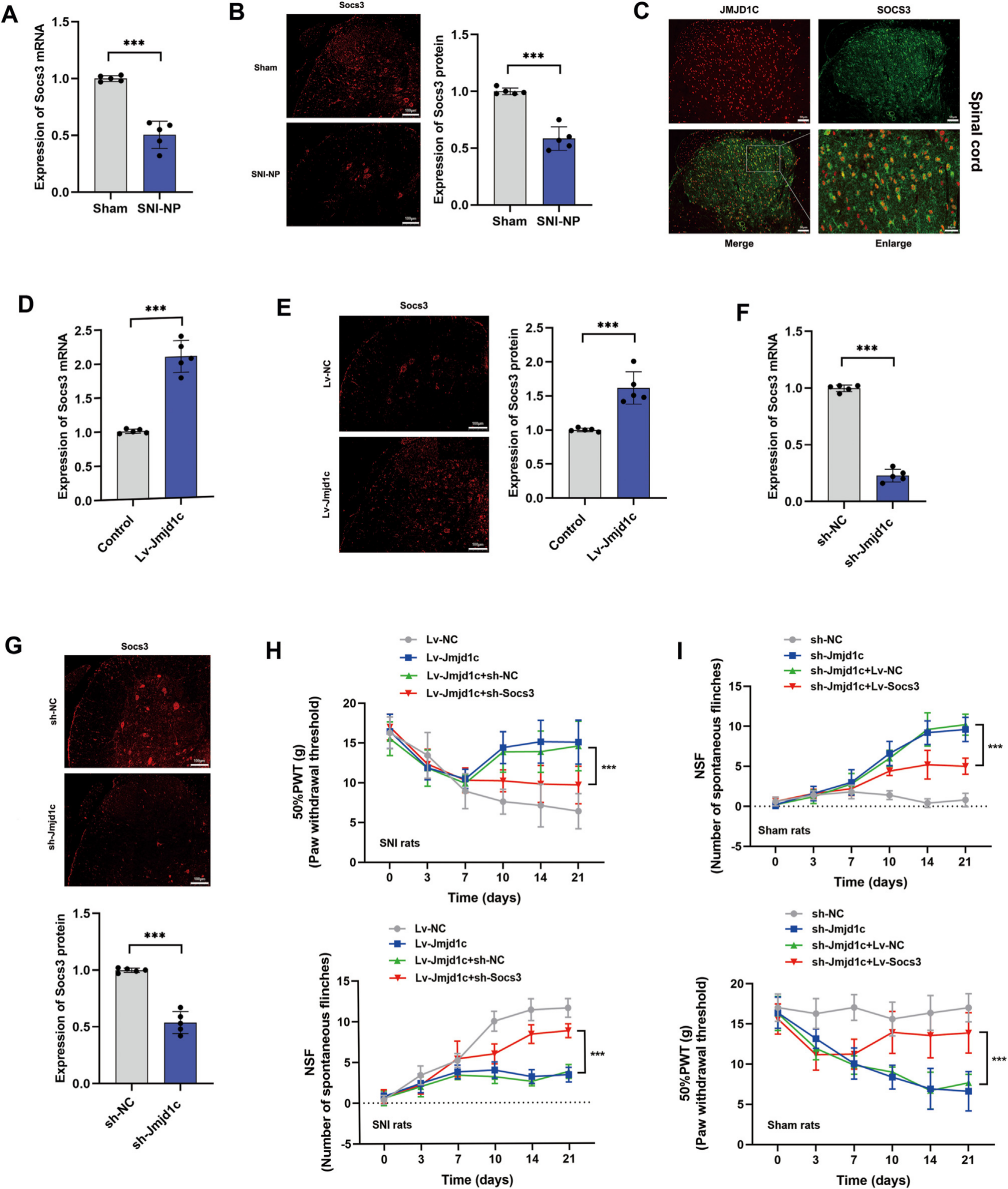
*Jmjd1c* suppresses NP via regulating *Socs3* expression. **(A)** Quantitative PCR showed that the relative expression of *Socs3* mRNA was decreased in SNI-NP rats. **(B)** Immunofluorescence detection showed that the protein expression of *Socs3* was decreased in SNI-NP rats. **(C)** Double-immunofluorescence assay showed the co-localization of *Jmjd1c* (Cy3-labelled, red) and *Socs3* (Alexa488-labelled, green) in spinal cord tissues. **(D)** The mRNA levels of *Socs3* after *Jmjd1c* overexpression in spinal cord tissues were determined by quantitative PCR and its expression was increased. **(E)** The protein levels of *Socs3* after *Jmjd1c* overexpression in spinal cord tissues were detected by immunofluorescence and the expression of *Socs3* protein was increased. **(F)** Quantitative PCR showed that the mRNA levels of *Socs3* in *Jmjd1c*-silencing NP rats were decreased. **(G)** Immunofluorescence detection showed that the *Socs3* protein levels in *Jmjd1c*-silencing NP rats were decreased. **(H)** PWT (upper) and NSF (lower) analyses proved that silencing of *Socs3* could effectively reverse the effects on PWT caused by *Jmjd1c* overexpression. **(I)** PWT (upper) and NSF (lower) analyses indicated that overexpression of *Socs3* could effectively reverse the effects on PWT caused by *Jmjd1c* knockout. The statistical results were presented as mean ± standard deviation. Student's *t*-test was used to determine the significance of the differences between the two groups. The asterisks indicate significance values (^∗∗∗^*P* < 0.001). NP, neuropathic pain; SNI, spared nerve injury; PWT, paw-withdrawal threshold; NSF, the number of spontaneous flinches; ELISA, enzyme-linked immunosorbent assay.

To verify the target of *Jmjd1c*, we screened the differentially expressed genes according to our RNA-seq data. Particularly, *Socs3* attracted our attention since it is down-regulated in the NP rat group and potently proved to suppress NP progression.^20^ Thus, we hypothesized that *Jmjd1c* may participate in the SNI regulation by regulating the expression of *Socs3*. To verify this hypothesis, we detected the expression of *Socs3*. The results indicated that both *Socs3* mRNA and protein expression were reduced in the spinal cord tissues of SNI-NP rats relative to sham rats (Fig. 6A, B). Double-immunofluorescence assay confirmed that *Socs3* was co-distributed with *Jmjd1c* in spinal cord tissues (Fig. 6C). When *Jmjd1c* was overexpressed by intrathecal injection of corresponding lentivirus vectors into SNI-NP rats, *Socs3* expression was notably increased in the spinal cord tissues (Fig. 6D, E). Consistently, *Sosc3* was silenced when sh-*Jmjd1c* was injected at both transcript and protein levels (Fig. 6F, G).

Next, we performed rescue experiments to verify whether *Jmjd1c* regulated the NP process via targeting *Socs3*. As shown in Figure 6H, overexpression of *Jmjd1c* suppressed NP, as evidenced by elevated PWT and suppressed NSF, however, this effect was dramatically attenuated by co-treatment with injection of *Socs3* silencing vectors. Consistently, enhanced *Socs3* significantly reversed the promoted NP status caused by *Jmjd1c* knockdown (Fig. 6I). The above data suggest that *Jmjd1c* exerted its anti-NP effect via targeting *Socs3* expression.

### *Jmjd1c* up-regulates *Socs3* by promoting H3K9 demethylation at *Socs3* promoter region

**Figure 7:**
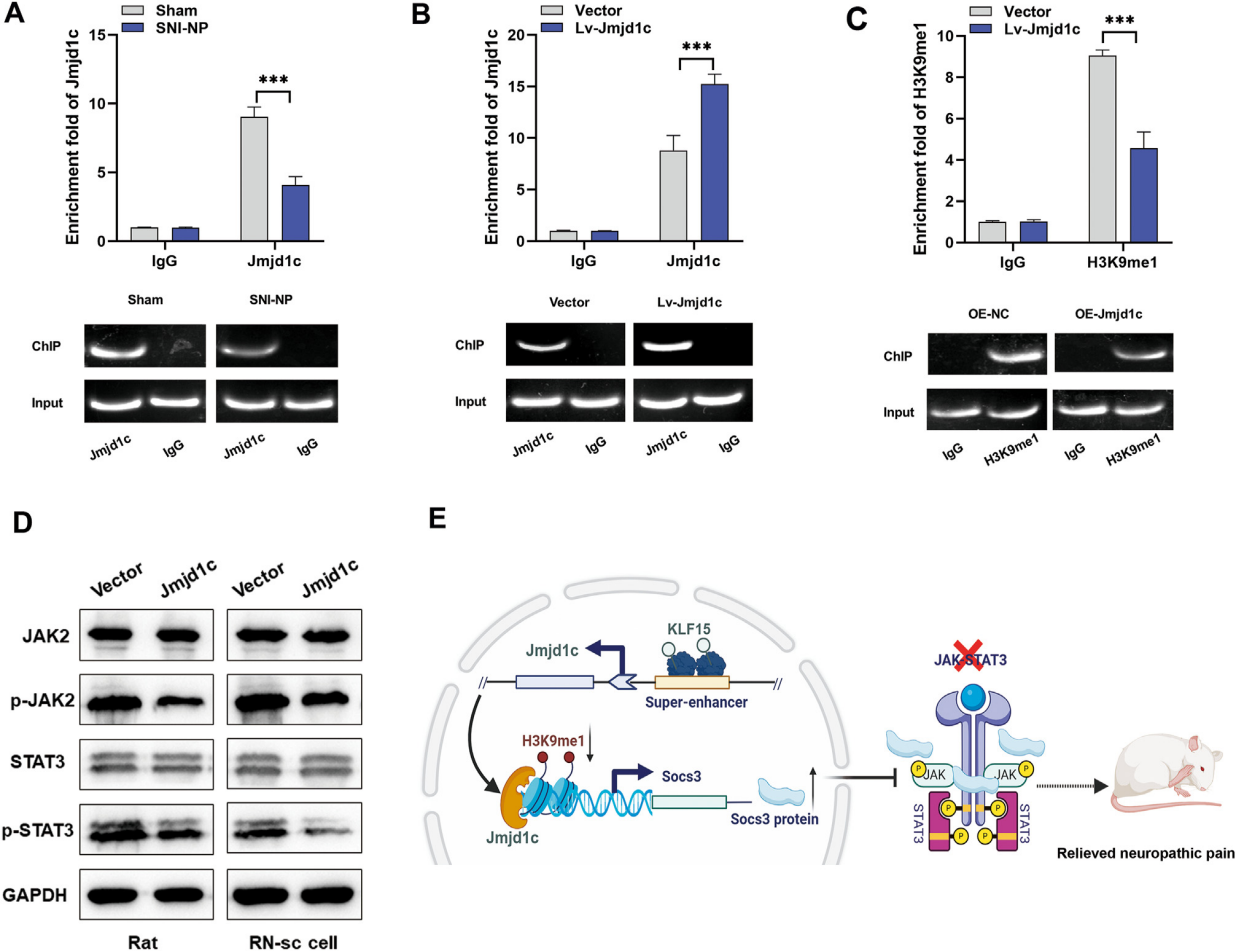
*Jmjd1c* inhibits NP progression via promoting H3K9 demethylation at the *Socs3* promoter region. **(A)** Chromatin immunoprecipitation analysis revealed that the enriched level of *Jmjd1c* in the promoters *Socs3* was significantly lower in SNI-NP rat tissues than those of sham rats. **(B)** Chromatin immunoprecipitation analysis showed that the enriched level of *Jmjd1c* at the *Socs3* promoter region was significantly higher in *Jmjd1c*-overexpressing rats. **(C)** Chromatin immunoprecipitation analysis indicated the enriched level of H3K9me1 at the *Socs3* promoter region was significantly lower in *Jmjd1c*-overexpressing rats. **(D)** Western blotting analysis showed the *Jmjd1c* suppressed the phosphorylation levels of *JAK2* and STAT3. **(E)** The schematic model for the mechanisms of *Jmjd1c* in NP progression. The statistical results were presented as mean ± standard deviation. Student's *t*-test was used to compare the means from two different data sets. The asterisks indicate significance values (^∗∗∗^*P* < 0.001). NP, neuropathic pain.

It has been previously reported that *Jmjd1c* can modulate the expression of target genes by mediating histone enrichment, such as through H3K9 demethylation.^21^ To verify whether *Jmjd1c* positively regulated *Socs3* expression via increasing the activity of H3K9 demethylation, we performed a ChIP analysis. As shown, the enriched level of *Jmjd1c* at *Socs3* promoter was significantly lower in NP rat tissues compared with sham ones (Fig. 7A). Meanwhile, the enrichment of *Jmjd1c* at *Socs3* promoter was significantly increased in rats injected with *Jmjd1c* vector (Fig. 7B). In addition, overexpression of *Jmjd1c* could suppress the enrichment level of H3K9me1 at *Socs3* promoter region (Fig. 7C). These data suggest that *Jmjd1c* may up-regulate *Socs3* expression via demethylation of H3K9 at promoter region.

It has been reported that *Socs3* suppressed the NP process via the inactivation of the *JAK/STAT3* pathway.^22^ To reveal whether *Jmjd1c* exerted its function through the regulation of *JAK2* and *STAT3* phosphorylation, we analyzed the expression level of related proteins. Our results showed that enhanced *Jmjd1c* suppressed the phosphorylation levels of *JAK2* and *STAT3* in RN-sc cells and SNI-rat (Fig. 7D).

## Discussion

Since NP seriously influences patients' quality of life, it is essential to understand the molecular mechanism of NP and develop effective therapeutic strategies. In this study, we focused on NP progression and explored how SE-associated epigenetic alterations were involved in the regulation of pain behavior. We identified that *KLF15* activated *Jmjd1c* transcription by binding to its SE, and *Jmjd1c* inhibited NP by positively regulating *Socs3* expression. Moreover, *Jmjd1c* suppressed the phosphorylation levels of *JAK2* and *STAT3* in the NP process (Fig. 7E). Our study demonstrated that *Jmjd1c* could act as an essential suppressor in NP via targeting *Socs3/JAK/STAT3* regulatory pathway.

NP remains one of the clinically challenging problems troubling human health, and the exact molecular and signaling mechanisms are not yet fully understood. SEs refer to tissue- and cell-specific genomic domains consisting of activated enhancer clusters, that recruit core regulatory circuitry transcription factors to mediate transcriptional dysregulation in many diseases, including cancer, alcoholic hepatitis, and cardiac regeneration.^23^, ^24^, ^25^ It has been reported that the activity of a spinal-specific SE, located upstream of the *Ntmt1* and *Prrx2* genes, was significantly increased in the dorsal horn of mice with NP. Mechanism experiment revealed that this spinal-specific SE is involved in the body's response to NP via driving expression of *Ntmt1* and *Prrx2*.^10^ But the regulatory role of SEs in NP progression is still poorly understood. Since the NP rat model provides a suitable research strategy for preclinical pain research.^26^ In our study, we first constructed the SNI-NP rat model and set out to define the enhancer landscapes in the spinal cord tissues of rats with NP. Interestingly, the abundance of H3K27ac-associated peaks was lower in the NP group compared with the sham group, we suspect that this may indicate reduced expression of key transcription factors that inhibit pain. Importantly, our data indicated that some well-known transcription factors, such as *KLF15*,^27^ *Myb44*,^28^ *Sox3*,^29^ and *HOXA1*,^30^ were found to be associated with NP-related SEs, supporting the functionality of SEs in NP. Complementary analysis of different platforms is essential to obtain a more comprehensive dataset related to the diseases.^31^ With integrative ChIP-seq and RNA-seq data, GO analysis revealed that the SE-associated genes may play essential roles in NP progression via affecting pain-related signaling pathways, such as “neuropeptide hormone activity”, “neuropeptide receptor binding”, and “neuropeptide receptor activity”, providing a theoretical basis for SEs to participate in the transcriptional activation of pain-related genes.

There is an urgent need to analyze the mechanism of NP progression and develop new targeted drugs. Previous studies have shown that *BRD4* plays a critical role in SE regulation and disease development,^32^, ^33^, ^34^ we tested directly whether *BRD4* inhibition influenced the activation of NP-related SEs in RN-sc cells. Strikingly, activation of *Jmjd1c* is dependent on *BRD4*, enhancing the therapeutic potential of *BRD4* inhibitors in treating NP patients. IL-6, TNF-α, and IL-1β are essential pain-related chemokines.^35^ Our analyses revealed that *Jmjd1c* was responsible for the induction of these pro-inflammatory factors. Previous studies found that SEs could recruit high levels of tissue-specific transcription factors and co-activators to regulate the expression of target genes.^36^ *KLF15*, a member of the zinc-finger family of transcription factors,^37^ may be involved in NP in mouse models.^38^ Based on our positional motif analysis, *KLF15* was found to activate *Jmjd1c* transcription by binding to its SE. According to this observation, we assume that Jmjd1c-SE inhibits NP progression by regulating key gene expression through the recruitment of *KLF15*, which might reveal one mechanism for how SEs participate in NP through transcription factors.

*Socs3* is one of the most essential negative feedback regulation transcription factors, inhibiting the inflammatory response and thus relieving inflammatory pain.^39^ Consistently, *Jmjd1c* was shown to interact with *Socs3*, and enhanced *Socs3* could significantly reverse the promoted NP status caused by *Jmjd1c* knockdown in our study. H3K9 histone demethylases are an important epigenetic regulator that controls chromatin structure and gene expression. It has been reported that *Jmjd1c* is a H3K9 demethylase,^40^ which is involved in the regulation of abnormal metabolic processes in different diseases such as leukemia and esophageal cancer.^41^^,^^42^ Therefore, it can be inferred that *Jmjd1c* plays an important role in the regulation of *Socs3* expression in the SNI-NP rat model. Subsequently, our results verified that *Jmjd1c* up-regulated *Socs3* via promoting the H3K9 demethylation of the *Socs3* promoter region both *in vivo* and *in vitro*. Due to the critical role of *JAK-STAT3* pathway in the spinal astrocyte proliferation and NP maintenance in rats,^13^ we detected the expression of related proteins and observed enhanced *Jmjd1c* suppressed the phosphorylation levels of *JAK2* and *STAT3* in the present study, which is consistent with previous reports that *Socs3* is an inhibitor of the *JAK/STAT3* pathway.^43^^,^^44^ Our data suggest a distinct role for *Jmjd1c* in the regulation of *Socs3* in modulating NP response in rats. It should be noted that there are still many other downstream target genes of *Jmjd1c* that are worthy of future investigation.

This study has certain limitations. Although we have preliminarily explored the functional role of Jmjd1c-SE and characterized its regulatory pathway *Jmjd1c/Socs3/JAK/STAT3*, these studies are insufficient. Therefore, in our subsequent research endeavors, it is necessary to further elucidate the combined effects of other pain-related SEs and their associated regulatory pathways in the pathogenesis and development of NP.

In conclusion, to our knowledge, we have mapped the SEs landscape for the first time in a rat model of NP. Additionally, we have also observed a reduction in SE-Jmjd1c in the NP model, and *Socs3* is an important upstream regulator of the *JAK/STAT3* signaling pathway, contributing significantly to NP progression. The findings of this preclinical research provide important evidence for the potential of *Jmjd1c* as a novel target for the treatment of NP. This study broadens our understanding of SEs, emphasizes the value of epigenomic analysis in rat models of NP, and further supports the need to identify SEs associated with NP to discover new therapeutic target genes.

## Ethics declaration

The study was rigorously examined and approved by the Animal Experiment Committee of The Second Hospital of Shandong University and strictly adhered to the standards set by the International Association for the Study of Pain.

## Funding

This work was supported by Shandong Provincial Natural Science Foundation (China) (No. ZR2021QH063).

## CRediT authorship contribution statement

**Le Zhang:** Data curation, Funding acquisition, Writing – original draft, Writing – review & editing. **Yan Xie:** Data curation, Methodology, Writing – original draft, Writing – review & editing. **Shun Wang:** Formal analysis, Resources. **Moxuan Gong:** Formal analysis, Investigation. **Zheping Chen:** Conceptualization, Data curation. **Chuanxin Wang:** Conceptualization, Project administration. **Peilong Li:** Project administration, Writing – original draft, Writing – review & editing.

## Conflict of interests

The authors declared no conflict of interests.
