# Supplementary material for: Enhancer profiling uncovers Jmjd1c as an essential suppressor in neuropathic pain by targeting Socs3
Source: Genes Dis. 2025 Jan 23;12(5):101545. doi: 10.1016/j.gendis.2025.101545 (PMC12143820; doi:10.1016/j.gendis.2025.101545)
Supplement: Multimedia component 1 [file mmc1.docx]

**Supplementary Materials**

**Supplementary figures**
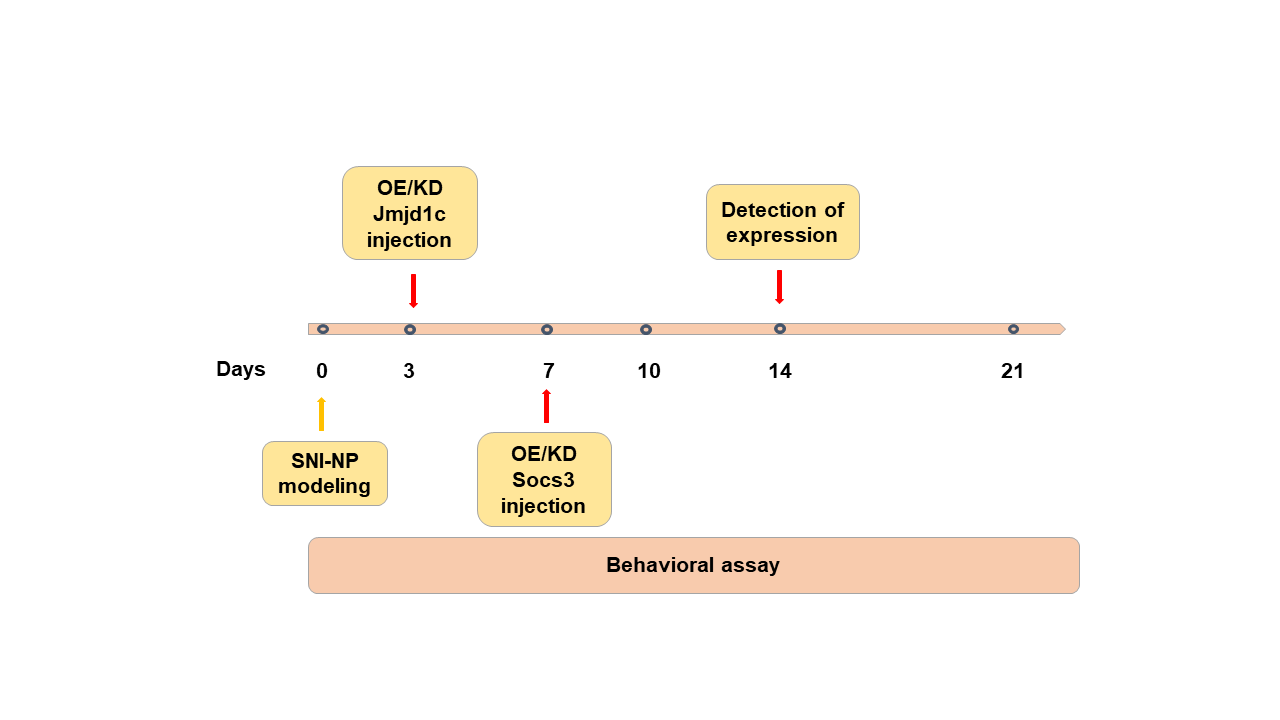


**Supplementary Fig.** **S1:** The flow chart of manipulations of detailed processing timeline of SNI-NP rats.


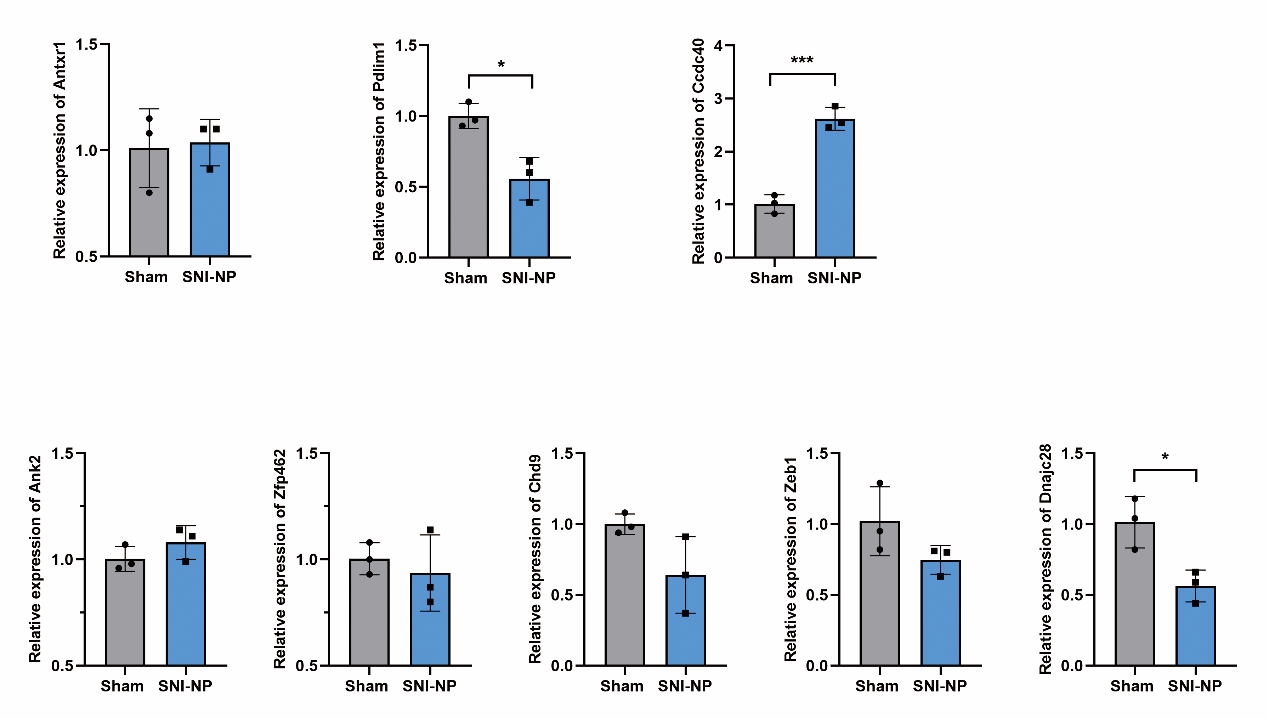


**Supplementary Fig. S2:** The expression of Antxr1, Pdlim1, Ccdc40, Ank2, Zfp462, Chd9, Zeb1, and Dnajc28 genes were detected by qPCR. *P<0.05, ***P<0.001.


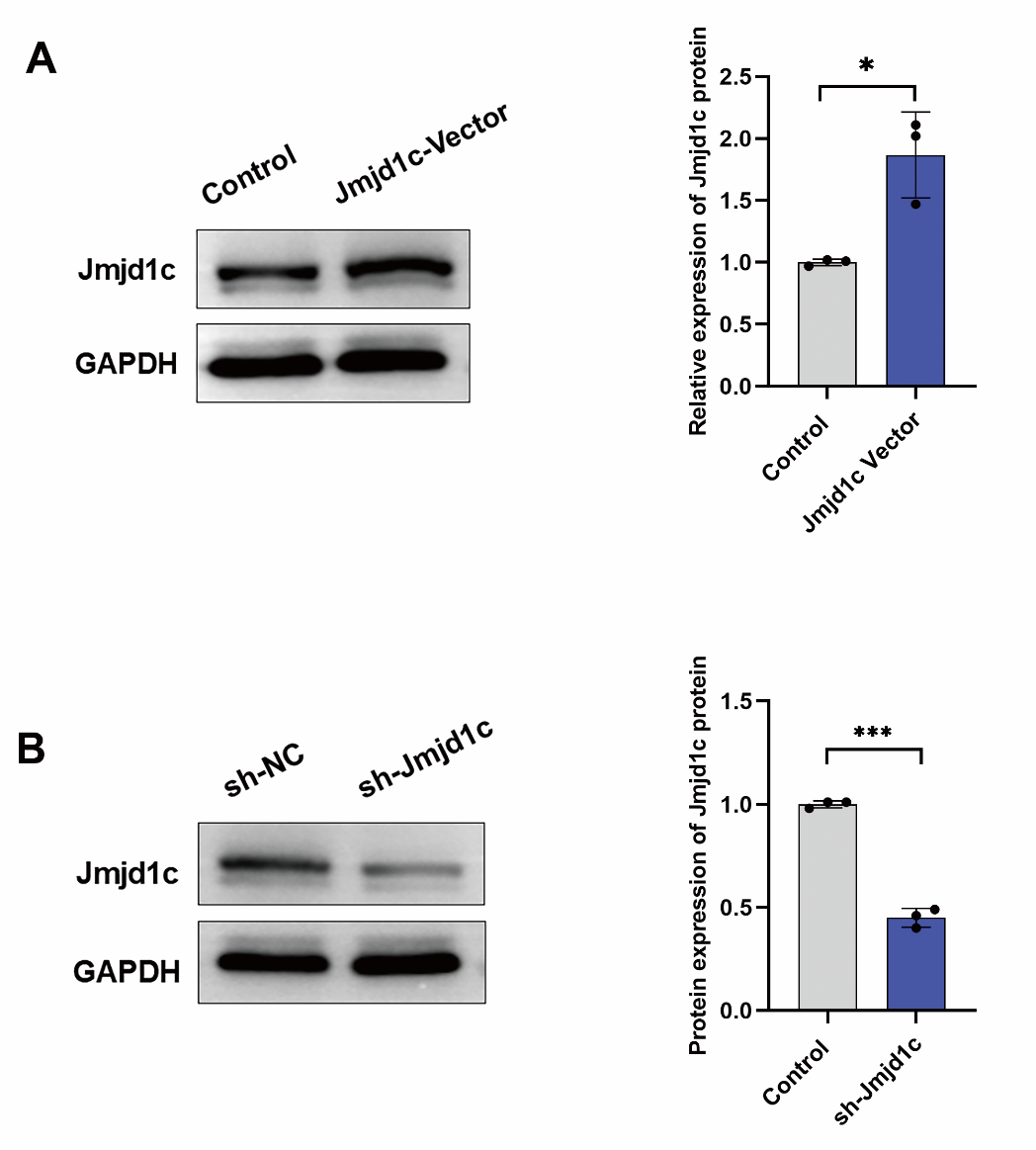


**Supplementary Fig. S3:** The expression of Jmjd1c protein in spinal cord of rats from respective groups were detected by western blot analysis. *P<0.05, ***P<0.001.
