# Supplementary material for: Enhancer profiling uncovers Jmjd1c as an essential suppressor in neuropathic pain by targeting Socs3
Source: Genes Dis. 2025 Jan 23;12(5):101545. doi: 10.1016/j.gendis.2025.101545 (PMC12143820; doi:10.1016/j.gendis.2025.101545)
Supplement: Multimedia component 2 [file mmc2.docx]

**Supplementary Table S1. Sequences for shRNAs**

| **Gene** | **Sequence (5’-3’)** |
| --- | --- |
| KLF15 | Sense CAGCAGCAGAACTTCTCAA |
|  | Antisense TTGAGAAGTTCTGCTGCTG |
| Jmjd1c | Sense GCCTCCACCTTTGATTAAA |
|  | Antisense TTTAATCAAAGGTGGAGGC |
| Socs3 | Sense GCTTTGACTGTGTACTCAA |
|  | Antisense TTGAGTACACAGTCAAAGC |

**Supplementary Table S2. Primers used for qPCR**

| **Gene** | **Sequence (5’-3’)** |
| --- | --- |
| Jmjd1c | Forward: AAACGAGCACTTCACGGTCTAT |
|  | Reverse: GGAAAGGATGCTGGCTCAAG |
| Antxr1 | Forward: TCCATCCTGGCGATTGCTCT |
|  | Reverse: TAAGAGGCGTCTACCGTGGG |
| Pdlim1 | Forward: AATCAAGGGCTGCGTAGACA |
|  | Reverse: AGCAGGTGAGGCGGTAAATG |
| Ccdc40 | Forward: GAAGCCCAGAAGATACCCTCAT |
|  | Reverse: GAGGGGAGGCAGAGTTGAAG |
| Chd9 | Forward: CAAACCAGACCCTTTAGTGCC |
|  | Reverse: ACTCTGTCCTTCCGTAGCCA |
| Ank2 | Forward: TCGCATCAAACAGGACAGCA |
|  | Reverse: GTGACTATTGGGCTGAAGGTTG |
| Dnajc28 | Forward: GACCGAGCAGGTGATGGAATA |
|  | Reverse: GGTTTTCCTTTCCCGCTGAG |
| Zeb1 | Forward: AGCACTATGACCCAGAGCACC |
|  | Reverse: ACAGAATCGGCAATCTTGGTGA |
| Zfp462 | Forward: CGAGGACAATGATGATGAGCC |
|  | Reverse: GTTGGTTGGTTTGGGATTAGGG |
| KLF15 | Forward: CTTCTCGTCACCGAAATGCC |
|  | Reverse: AAATCCAAGATGCTGCCCTG |
| Socs3 | Forward: TCTTTACCACCGACGGAACC |
|  | Reverse: CGACAAAGATGCTGGAGGGT |
| GAPDH | Forward: CATGACCACAGTCCATGCCA |
|  | Reverse: CAGGGATGATGTTCTGGGCT |

**Supplementary Table S3. Detailed information of antibodies used in Immunofluorescence staining and ChIP analysis**

| **Antibody** | **Source** | **Catalog number** | **Working dilution** |
| --- | --- | --- | --- |
| anti-Jmjd1c antibody for immunofluorescence | Abclonal, China | A20153 | 1:200 |
| anti-NeuN antibody | Abcam, USA | ab104224 | 1:200 |
| anti-GFAP antibody | Cell Signal Technology, USA | 3670S | 1:200 |
| anti-IBA1 antibody | Abcam, USA | ab5076 | 1:200 |
| anti- Socs3 antibody | Abclonal, Wuhan, China | A0694 | 1:200 |
| Goat anti-rabbit IgG labeled with Alexa Fluor 488 | Servicebio, China | GB25303 | 1:500 |
| Goat anti-rabbit IgG labeled with Alexa Fluor 594 | Servicebio, China | GB28301 | 1:500 |
| anti-Jmjd1c antibody for ChIP | EMD Millipore, USA | 17-10262 | 1:100 |
| anti-H3K9me1 antibody for ChIP | Abcam, USA | Ab9045 | 1:100 |
| anti-KLF5 antibody for ChIP | Abcam, USA | Ab277775 | 1:100 |

**Supplementary Table S4. Detailed information of antibodies used in Western blot**

| **Antibody** | **Source** | **Catalog number** | **Working dilution** |
| --- | --- | --- | --- |
| anti-GAPDH antibody | Cell Signal Technology, USA | 3670S | 1:1000 |
| anti-Jmjd1c antibody | Abclonal, China | A20153 | 1:200 |
| anti-JAK2 antibody | HUABIO, China | ET1607-35 | 1:500 |
| anti-P-JAK2 antibody | HUABIO, China | ET1607-34 | 1:500 |
| anti-STAT antibody | Cell Signal Technology, USA | 9139S | 1:1000 |
| anti-P-STAT antibody | Cell Signal Technology, USA | 9145T | 1:2000 |
| goat anti-rabbit IgG | Jackson | 111-035-144 | 1:5000 |
| goat anti-mouse IgG | Jackson | 115-005-003 | 1:5000 |
